# Supplementary material for: Rhizosphere Microbiome and Phenolic Acid Exudation of the Healthy and Diseased American Ginseng Were Modulated by the Cropping History
Source: Plants (Basel). 2023 Aug 19;12(16):2993. doi: 10.3390/plants12162993 (PMC10459672; doi:10.3390/plants12162993)
Supplement: Supplementary file 1 [file plants-12-02993-s001.zip › plants-2546064-supplementary.pdf]

Table S1. Relative abundance of fungi in the rhizosphere soil of diseased and healthy American ginseng at the phylum level

Phylum	Relative abundance (%)							
	1Y-H	1Y-D	2Y-H	2Y-D	3Y-H	3Y-D	4Y-H	4Y-D
Ascomycota	81.2	80.05	71.97	83.93	83.19	86.47	75.27	63.35
Basidiomycota	8.77	8.39	10.48	4.9	4.69	2.48	6.28	2.73
Mortierellomycota	1.91	1.76	2.4	2.05	3.08	1.26	3.14	0.88
Mucoromycota	1.39	0.45	5.39	1.32	2.77	0.33	11.15	2.65
other	0.04	0	0.06	0.08	0.22	0.1	0	0
unclassified	6.69	9.36	9.69	7.72	6.05	9.35	4.25	30.39

Table S2. Relative abundance of bacteria in the rhizosphere soil of diseased and healthy American ginseng at the phylum level

Phylum	Relative abundancen (%)							
	1Y-H	1Y-D	2Y-H	2Y-D	3Y-H	3Y-D	4Y-H	4Y-D
Proteobacteria	35.1	38.58	38.95	43.69	39.53	37.63	34.27	48.83
Actinobacteria	33.74	28.27	28.22	26.67	20.73	19.07	24.12	24.26
Acidobacteria	9.2	11.19	13.62	12.55	14.47	17.29	12.3	7.99
Firmicutes	11.63	10.94	8.77	7.02	12.08	11.04	19.31	7.85
Chloroflexi	5.6	6.05	5.74	4.87	6.54	6.88	5.45	3.21
Bacteroidetes	0.97	1.37	0.95	2.1	2.08	3.09	1.06	5.5
Gemmatimonadetes	1.64	1.74	1.66	1.14	1.79	1.69	1.64	0.79
Other	2.14	1.88	2.09	1.96	2.77	3.31	1.83	1.59

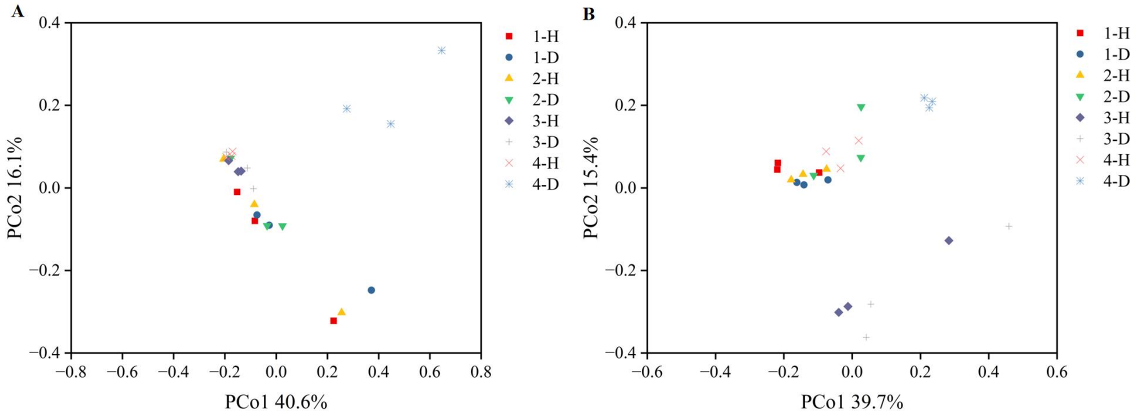

Fig S1. Principal component analysis of American ginseng rhizosphere fungi (A) and bacteria (B) in soils with the healthy (H) and diseased (D) American ginseng in 1-4 years of continuous monocropping (1Y, 2Y, 3Y and 4Y).
